# Temporal characterization of femtosecond laser pulses using tunneling ionization in the UV, visible, and mid-IR ranges

**DOI:** 10.1038/s41598-019-52237-y

**Published:** 2019-11-05

**Authors:** Wosik Cho, Sung In Hwang, Chang Hee Nam, Mina R. Bionta, Philippe Lassonde, Bruno E. Schmidt, Heide Ibrahim, François Légaré, Kyung Taec Kim

**Affiliations:** 1 Center for Relativistic Laser Science, Institute for Basic Science, Gwangju, 61005 Korea; 2 Department of Physics and Photon Science, Gwangju Institute of Science and Technology, Gwangju, 61005 Korea; 3 Institut National de la Recherche Scientifique, Centre Énergie Matériaux et Télécommunications, 1650 Boulevard Lionel-Boulet, Varennes, Quebec J3X 1S2 Canada; 4 Few-cycle Inc., 2890 Rue de Beaurivage, Montreal, Quebec H1L 5W5 Canada; 5 Department of Electrical Engineering and Computer Science, Massachusetts Institute of Technology, 77 Massachusetts Avenue, Cambridge, MA 02139 USA

**Keywords:** Optics and photonics, Ultrafast lasers

## Abstract

To generalize the applicability of the temporal characterization technique called “tunneling ionization with a perturbation for the time-domain observation of an electric field” (TIPTOE), the technique is examined in the multicycle regime over a broad wavelength range, from the UV to the IR range. The technique is rigorously analyzed first by solving the time-dependent Schrödinger equation. Then, experimental verification is demonstrated over an almost 5-octave wavelength range at 266, 1800, 4000 and 8000 nm by utilizing the same nonlinear medium – air. The experimentally obtained dispersion values of the materials used for the dispersion control show very good agreement with the ones calculated using the material dispersion data and the pulse duration results obtained for 1800 and 4000 nm agree well with the frequency-resolved optical gating measurements. The universality of TIPTOE arises from its phase-matching-free nature and its unprecedented broadband operation range.

## Introduction

The temporal characterization of a laser pulse is often the first essential step for studying ultrafast light-matter interactions. Many characterization techniques have already been developed, which can be classified into two categories depending on their measurement schemes. The first approach uses the optical response of a nonlinear material (for example, second harmonic generation in a nonlinear crystal) and includes techniques known as FROG^1^, SPIDER^2^, D-SCAN^3^, and others^4,5^. These methods are widely used in many applications because they can be implemented with a simple apparatus. However, they can only be applied to a spectral range that is limited by the phase-matching bandwidth of the specific nonlinear interaction employed^6,7^. Although some of these methods can cover a spectrum over one octave^8,9^, it is in general difficult to apply these techniques over a multioctave spectral range. The second approach uses an ultrafast temporal gate, such as an attosecond X-ray pulse, to directly sample a light field. The attosecond streak camera^10^, petahertz optical oscilloscope^11^ and ARIES^12^ fall into this category. These methods support the complete temporal characterization of a laser field for a broad spectral range, including the UV, visible and IR ranges^13–15^; however, they require complex equipment in vacuum. Consequently, there has been a high demand for an easy temporal characterization method that is applicable over a broad spectral range without the need for a vacuum environment.

Recently, a new pulse characterization technique called tunneling ionization with a perturbation for the time-domain observation of an electric field (TIPTOE) was introduced^16^. TIPTOE directly samples the electric field of a weak pulse that perturbs the ionization induced by a sufficiently strong laser pulse. Since the technique utilizes the extreme nonlinearity of ionization, it can be applied in air or with other gaseous molecules. The technique does not require a vacuum environment and is free from damage limitations of the nonlinear medium. Most importantly, it is expected that the method will be applicable over a broad spectral range, since tunneling ionization universally occurs regardless of the wavelength of a laser pulse. However, the experimental verification of the applicability for a broad wavelength range has not been demonstrated. TIPTOE has been tested only for single-cycle and few-cycle laser pulses in the wavelength range of 400–1000 nm^16,17^.

In this work, we demonstrate the universality of TIPTOE by applying it over a broad wavelength range from the UV range to the IR range in the chirped, multicycle regime and discuss the underlying basic theory. Several fundamental aspects are addressed for the first time both theoretically and experimentally. The validity of TIPTOE is tested by solving the time-dependent Schrödinger equation (TDSE) for various cases. We also perform experiments at wavelengths of 266, 1800, 4000, and 8000 nm. The experimental results obtained at 1800 and 4000 nm are compared with the results obtained using the FROG technique.

## Results

### TIPTOE theory in the multicycle regime

An arbitrary time-dependent laser field can be measured in the time domain when a subcycle temporal gate is available. In TIPTOE, subcycle ionization in an intense laser pulse provides such a temporal gate. Two laser pulses, referred to as the fundamental (E_F_) and the signal (E_S_), are used. The fundamental pulse is strong enough to induce ionization, while the signal pulse is too weak by itself to cause ionization. It, however, interferes with the fundamental pulse, resulting in a modulation of the ionization yield that contains information of the temporal profile of the signal pulse.

**Figure 1:**
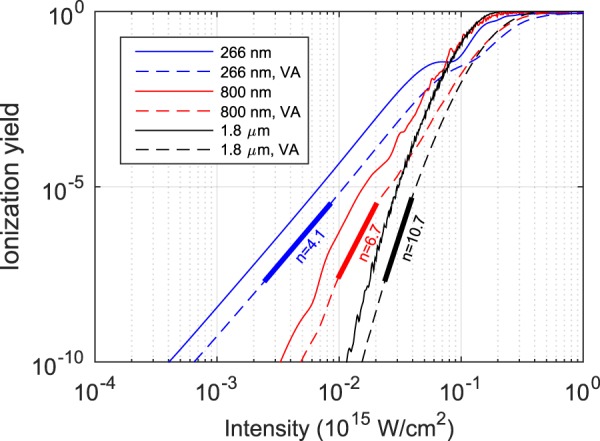
Ionization yield calculated at different laser intensities by solving the TDSE in 1D with a soft-core potential (Ip = 12.07 eV) and 25 fs laser pulses at three wavelengths of 266, 800, and 1800 nm. The single atom ionization yields are given as solid lines, while the ones obtained for the focal volume (volume average, VA) are plotted with dashed lines. The nonlinear coefficient *n* is obtained by a linear fit (short thick lines).

An accurate description of the ionization in a multicycle laser field requires the consideration of both the amplitude and the phase of an electron wavepacket created by either multiphoton absorption or tunneling. The interference of electron wavepackets created at different half cycles of the laser pulse should be taken into account when modeling the ionization^18^. The ionization yields of single atoms, calculated by solving the TDSE, are shown in Fig. 1. The ionization yields increase with intensity, but they also exhibit wiggles due to interference effects. These are more pronounced for a longer wavelength laser with high intensity. Therefore, an accurate theoretical model is required for modeling the ionization yield of a single atom.

In TIPTOE, the ionization yield is simply modeled as the integration of the ionization rate, neglecting interference effects. As we are interested in the total ionization yield obtained over a volume where individual atoms experience different intensities, such interference effects are averaged out. The ionization yield obtained for the focal volume follows a smooth increase with increasing laser intensity, as shown in Fig. 1. Thus, the total ionization yield obtained from a focal volume can be approximated by the integration of the instantaneous ionization rate w(*E*) as

1 $$N(\tau )={\int }_{-\infty }^{+\infty }\,w[{E}_{F}(t-\tau )+{E}_{S}(t)]dt.$$

In Eq. (1), we also neglect ground state depletion, excitation and ionization from the excited states. Although Eq. (1) is derived under certain approximations, it is accurate enough for the description of the ionization yield modulation shown in the following.

The ionization rate *w*(*E*) can be obtained using various ionization models such as the ADK^19^, PPT^20^ and Yudin-Ivanov models^21^. It should be noted that TIPTOE does not rely on a particular ionization model as long as the extreme nonlinearity of ionization is properly modeled. Furthermore, the TIPTOE method is not restricted to being implemented in the tunneling regime [as opposed to what its name ‘TIPTOE (tunneling ionization …)’ may suggest]. Therefore, for the sake of simplicity, the ionization rate defined as *w*(*E*) = *I*^*n*^ = *E*^2*n*^ can be used. Here, the integer *n* is the nonlinearity coefficient, which can be estimated from the slope of the ionization curve in Fig. 1.

For the description of the ionization yield modulation, we also assume that the temporal profiles of the fundamental and signal pulses are identical (i.e., *E*(*t*) = *E*_*F*_(*t*) = *E*_*S*_(*t*)/*r*, with *r* being the amplitude ratio between the two pulses). For *r*≪1, the modulation of the ionization yield can be expanded as *N*(*τ*) = *δ*_*N*_^(0)^ + *δ*_*N*_^(1)^ + *δ*_*N*_^(2)^ + …. The zeroth order *δ*_*N*_^(0)^ is a constant. It is the ionization yield obtained without the signal field. The first order δ_N_^(1)^ that contains the amplitude and phase information of the signal pulse can be written as

2 $${\delta }_{N}^{(1)}(\tau )=2nr\int E{(t-\tau )}^{2n-1}\,E(t)dt.$$

As we increase the amplitude ratio *r*, the higher orders can be included in the ionization yield modulation by which the modulation becomes asymmetric. However, this asymmetry can be removed by frequency filtering.

### Frequency response of the TIPTOE measurements in the multicycle regime

The first derivative of the ionization [*dw*/*dE* = *E*(*t* − *τ*)^2*n*−1^] plays the role of the temporal gate. The effect of the temporal gate differs depending on the temporal shape of the pulse. If the duration is extremely short and the carrier envelop phase (CEP) of the pulse is zero (i.e. cosine like pulse), the ionization occurs in a single half optical cycle. The derivative (*dw*/*dE* = *E*(*t* − *τ*)^2*n*−1^) behaves like a delta function. The electric field can be directly obtained from the modulation of the ionization yield as δ_N_(*τ*) ∝ *E*(*τ*). If the pulse duration is long (i.e., multicycle), ionization occurs over multiple half optical cycles over which the signal field is sampled. In this case, the ionization yield modulation may not directly represent the temporal profile of the signal pulse.

Since the expression for the ionization yield modulation δ_N_^(1)^ in Eq. 2 is the cross-correlation of the two functions, *E*(*t* − *τ*)^2*n*−1^and *E*(*t*), the expression can be written with the cross-correlation theorem (or convolution theorem) in the frequency domain as

3 $$ {\mathcal F} \{{\delta }_{N}^{(1)}(t)\}\propto  {\mathcal F} {\{E{(t)}^{2n-1}\}}^{\ast }\cdot  {\mathcal F} \{E(t)\}.$$

Here, ${\mathcal F}$ denotes the Fourier transform. ${\mathcal F} {\{E{(t)}^{2n-1}\}}^{\ast }$ is the frequency response of the TIPTOE measurement that determines the relation between the ionization yield modulation and the original field. The modulation of the ionization yield *δ*_*N*_(*t*) may differ from the signal field *E*(*t*) depending on the frequency response ${\mathcal F} {\{E{(t)}^{2n-1}\}}^{\ast }$. Therefore, to extract the signal pulse from the modulation of the ionization yield, the effect of the frequency response should be properly considered.

### General property of the frequency response function

The multiplication of the frequency response function ${\mathcal F} {\{E{(t)}^{2n-1}\}}^{\ast }$ affects both in amplitude and phase. It is clear from Eq. 2 that the CEP information of the signal pulse is canceled out due to the multiplication of the frequency response function if the fundamental and signal pulses have an identical temporal profile. In order to measure the temporal profile of the laser pulse including the CEP, the CEP of the fundamental pulse should be set so that a single dominant ionization occurs in a half optical cycle. This condition can be found by using an additional second harmonic pulse^16^. In this work, however, we measure multi-cycle laser pulses for which the CEP is not an important parameter. Therefore, we use an inline interferometer in which the fundamental and signal pulses co-propagate and have identical temporal profiles. The CEP of the laser pulse is not stabilized.

**Figure 2:**
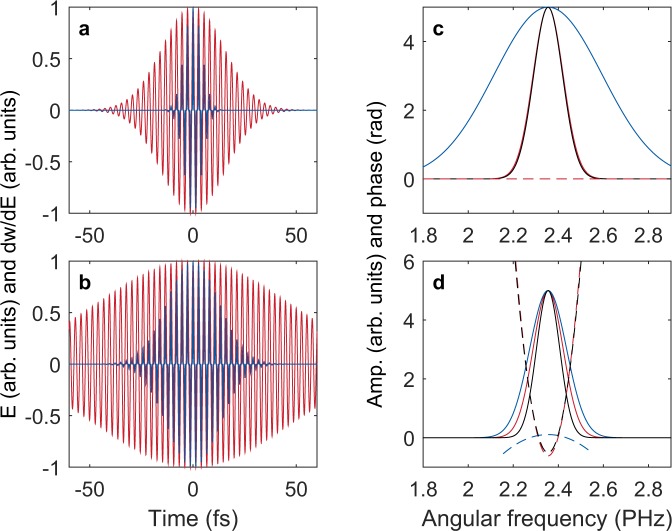
Frequency response of the TIPTOE measurement in the multicycle regime. The frequency responses are obtained using the ionization model (*w* = *I*^*n*^) with a nonlinearity coefficient n=7 and for fundamental pulses having a transform-limited duration of 25 fs. (**a**,**b**) The first-order derivative of the ionization rate (dw/dE, blue line) is calculated using a chirp-free 25 fs pulse in (**a**) and using a chirped 75 fs pulse with a GDD of 640 fs^2^ in (**b**). (**c**,**d**) The amplitude (solid lines) and phase (dashed lines) of the signal pulse spectrum ${\mathcal F} \{E(t)\}$ (red lines) and the frequency response ${\mathcal F} {\{E{(t)}^{2n-1}\}}^{\ast }$ (blue lines) are given in (**c**) and (**d**) for (**a**,**b**), respectively. The black lines show the amplitude and phase of the ionization yield spectrum ${\mathcal F} \{{\delta }_{N}(t)\}$.

The bandwidth of the ionization yield modulation can be narrower than the bandwidth of the signal pulse due to the multiplication of the frequency response function ${\mathcal F} {\{E{(t)}^{2n-1}\}}^{\ast }$. To understand this effect, we compare two cases calculated using transform-limited and chirped Gaussian pulses. The laser pulse of the transform-limited Gaussian pulse is shown in red in Fig. 2a, together with the first-order derivative of the ionization modulation [*E*(*t*)^2*n*−1^] in blue. The duration of the first-order derivative is only 28% of the pulse due to the high nonlinearity (*n* = 7) of the ionization. In this case, the amplitude of the frequency response near the central frequency is broader than that of the signal pulse spectrum, and the phase is flat, as shown in Fig. 2a.

The spectral bandwidth of the modulation (${\mathcal F} \{{\delta }_{N}(t)\}$) can be analytically calculated for the transform-limited Gaussian pulse as $\Delta {\rm{\omega }}\sqrt{(2n-1)/(2n)}$, where Δω is the bandwidth of the signal pulse. When *n* = 7, the spectral bandwidth of ${\mathcal F} \{{\delta }_{N}(t)\}$ is only slightly reduced. The spectral bandwidth is 96% of the bandwidth of the signal pulse, as shown by the black and red lines in Fig. 2c. Thus, the effect of the frequency response would be negligible when the pulse duration is close to transform-limited. In this case, the ionization yield modulation *δ*_*N*_(*t*) is a very good approximation of the signal pulse *E*(*t*).

If the pulse is chirped, the effect of the frequency response becomes significant. As a second example, the ionization rate is calculated using a chirped Gaussian pulse whose duration (75 fs) is three times longer than the transform-limited duration (25 fs), as shown in Fig. 2b. The duration of the pulse is estimated by the full width at half maximum (FWHM). The duration of the first-order derivative [*E*(*t*)^2*n*−1^] is now comparable with the transform-limited duration, as shown in Fig. 2b. The bandwidth of the frequency response is still broader than the signal pulse spectrum. However, the spectral bandwidth of ${\mathcal F} \{{\delta }_{N}(t)\}$ is only 82% of the signal bandwidth, as shown in Fig. 2d. The spectral phase of the frequency response is also slightly curved with an opposite sign. The spectral phase of the modulation (${\mathcal F} \{{\delta }_{N}(t)\}$) slightly differs from the spectral phase of the signal pulse. Therefore, the ionization yield modulation does not directly represent the original pulse. An appropriate reconstruction process to find the signal pulse from the ionization yield modulation is required.

### Reconstruction of the signal pulse

The frequency response of the TIPTOE measurement can be corrected through a few different approaches. In this work, we use a very simple approach in which we assume that the spectral amplitude of the original pulse is known (e.g., due to a measurement of the spectral intensity). First, an approximated signal pulse *E*^′^(*t*) is obtained by taking an inverse Fourier transform as $E^{\prime} (t)={ {\mathcal F} }^{-1}\{\tilde{A}(\omega )\exp [i\tilde{\varphi }(\omega )]\}$ using the amplitude *A*(*ω*) obtained from the spectrum and the phase $\tilde{\varphi }(\omega )=Arg[{\tilde{\delta }}_{N}(\omega )]$ obtained from the ionization yield modulation. The phase of the signal pulse is then corrected from the frequency response calculated using the approximated signal pulse. (i.e., ${\tilde{\varphi }}_{corr}(\omega )={\rm{Arg}}\{ {\mathcal F} {[E\text{'}{(t)}^{2n-1}]}^{\ast }\})$. Finally, the signal pulse is reconstructed using ${E}_{s}(t)={ {\mathcal F} }^{-1}\{\tilde{A}(\omega )\exp \{i[\tilde{\varphi }(\omega )-{\tilde{\varphi }}_{corr}(\omega )]\}\}.$

It should be noted that the reconstruction can only be applied when the input pulse is not much longer than the transform-limited duration. If the pulse is too long, ionization occurs over many multiple half optical cycles over which the signal field is sampled. This multicycle effect results in a narrow spectrum of ${\mathcal F} \{{\delta }_{N}(t)\}$, as shown in Fig. 2d, which sets the fundamental limitation of the TIPTOE measurement. The spectral amplitude itself can be recovered using the separately measured spectrum; however, the spectral phase information on both sides of the spectrum becomes inaccurate when the spectral amplitude of ${\mathcal F} \{{\delta }_{N}(t)\}$ is small. Therefore, the TIPTOE measurement becomes inaccurate if the pulse is much longer than the transform-limited duration.

**Figure 3:**
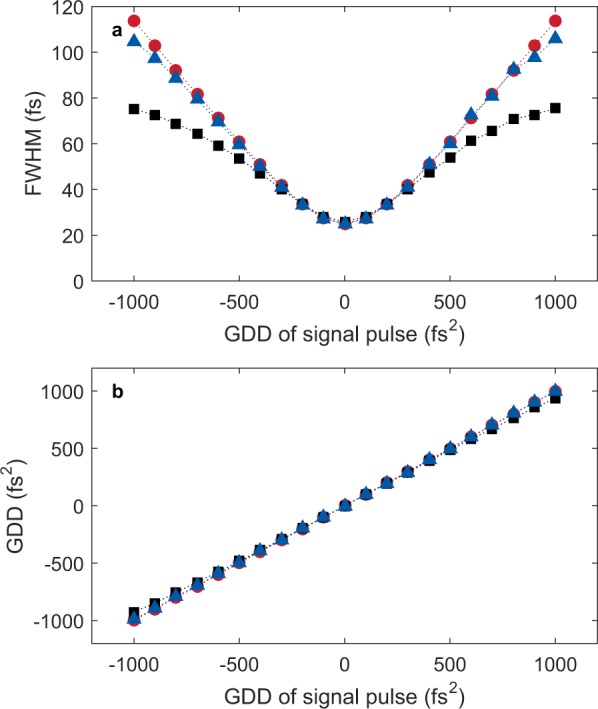
Durations and GDDs calculated for chirped Gaussian pulses by solving the TDSE model. (**a**,**b**) The duration in the FWHM and the GDD are shown in (**a**,**b**) for the original signal pulse (red circles), the ionization yield modulation (black squares), and the reconstructed pulse (blue triangles).

To test the accuracy of the TIPTOE measurement quantitatively, we perform TDSE (1d) calculations in which a soft-core potential with the ionization potential of O_2_ (12.07 eV) is used. We assume that the temporal shapes of the two pulses (fundamental and signal pulses) are identical. The peak intensity of the fundamental pulse is 1 × 10^13^ W/cm^2^, and the intensity of the signal pulse is 1 × 10^10^ W/cm^2^ at a wavelength of 800 nm. The ionization yield is calculated from atoms distributed near the focus. The total ionization yield is obtained by integrating over the focal volume. A chirped Gaussian pulse is examined whose transform-limited duration (τ_TL_) is 25 fs. We obtained the ionization yield modulation for signal pulses with a GDD from −1000 fs^2^ to 1000 fs^2^. The reconstruction results are summarized in Fig. 3. The duration of the ionization yield modulation only shows good agreement with the duration of the original pulse (τ) for low GDD values. The error becomes large for high GDD values, as shown in Fig. 3a. However, the duration of the reconstructed pulse is accurate even for high GDD values with an error below 5% for τ < 4τ_TL_. The phase of the reconstructed signal pulse is very accurate, as shown in Fig. 3b, with an error below 3% for τ < 4τ_TL_. These results indicate that, as expected, the reconstruction error increases as the pulse duration increases, and the reconstruction errors for the duration and GDD remain below 5% when τ < 4τ_TL_.

Thus far, we have discussed the characteristics of the frequency response of TIPTOE measurements for a single Gaussian pulse. We now study advanced temporal structures. In general, the existence of weak pre- and postpulses will not affect the accuracy of the reconstruction of a TIPTOE measurement due to a high nonlinearity, unless there is a comparable peak intensity with the main pulse. Thus, the frequency response in a TIPTOE measurement is determined only by the duration of the main pulse that contributes to ionization.

**Figure 4:**
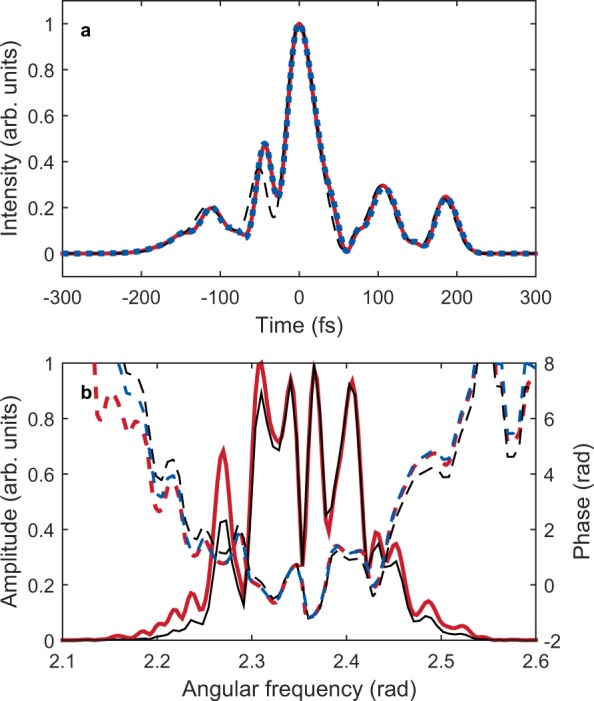
Reconstruction of a complex signal pulse. (**a**) Intensity profile of the original signal pulse (red line), the ionization yield modulation (black dashed line), and the reconstructed signal pulse (blue dashed line). (**b**) Spectral amplitude (solid lines) and phase (dashed lines) of the original signal pulse (red), the ionization yield modulation (black), and the reconstructed signal pulse (blue line).

To test the accuracy of the reconstruction for such a complex temporal structure, a test pulse is created by adding a pre- and postpulse to the main pulse, as shown in Fig. 4a. The intensity profile of the ionization yield modulation already shows good agreement with the original pulse. The spectral amplitude of the ionization yield modulation is slightly narrower than the original pulse, as expected (Fig. 4b), because the duration of the main pulse (42.7 fs) is slightly longer than the transform-limited duration (25.1 fs). When the amplitude and phase are corrected, the reconstructed pulse shows a temporal profile identical to the original pulse with a duration of 42.9 fs, as shown in Fig. 4a. Therefore, the TIPTOE method is generally applicable in the multicycle regime.

The reconstruction method described here would not work if multiple pulses contribute to ionization. To handle a more general condition, a reconstruction algorithm that directly finds a solution of Eq. 1 should be developed. However, the development of such an algorithm and its stability and accuracy tests are beyond the scope of the current work. We will discuss these improvements in future works. Therefore, we applied the simple reconstruction method described here for the limited range of the pulse durations (e.g., τ < 4τ_TL_) to show the applicability of the TIPTOE method in the multicycle regime for a broad range of wavelengths.

## Experimental results

**Figure 5:**
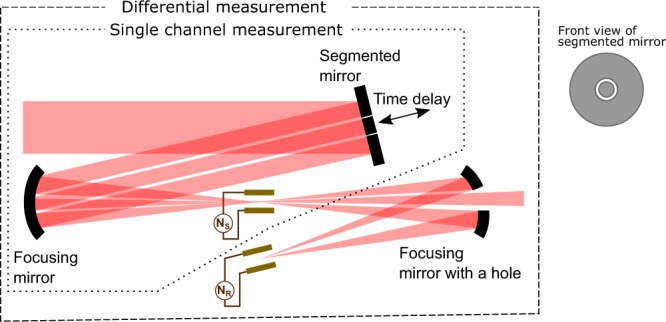
Inline experimental setup for the TIPTOE measurement. A segmented mirror separates the input laser beam into two pulses. Both beams are focused in the middle of two metallic plates connected to the current measurement device. After that, the inner beam is dumped by a holey mirror, and the outer beam is refocused for the reference current measurement.

The experimental demonstration of the TIPTOE method in the multicycle regime was performed using the inline experimental setup depicted in Fig. 5. A segmented mirror, which consists of two concentric mirrors, separates the input laser beam into two beams. The beam reflected by the outer annular mirror is more tightly focused at the focus than the beam reflected by the inner mirror. Thus, the outer beam is the fundamental beam, which ionizes air molecules, and the inner part corresponds to the signal beam to be characterized. We found that the shape of the fundamental beam at the focus is important for the accurate measurement. It should be a well-defined single beam. If it produces multiple foci, ionization yield modulation may not represent the signal beam correctly. The intensity ratio between the two beams can be adjusted by the power and the size of the input beam. Their relative time delay is controlled by a piezo transducer attached to the inner mirror. The ionization yield (N_S_) is measured by two metallic plates connected to a current measurement device. The ionization yield modulation (δ_N_) is estimated by the ratio of the ionization yield with its mean value (*N–*_*S*_) and subtracting 1 (i.e., δ_N_ = *N*_*S*_/*N–*_*S*_ − 1). The basic operation of TIPTOE requires these three parts (the segmented mirror, focusing mirror, and current measurement), which we call a single channel measurement.

For an unstable laser source, the ionization yield measurement is often very noisy due to the intrinsic power fluctuations. In such a case, an additional device to measure a reference current can be added, using a mirror with a through hole. This mirror is used to dump the signal (inner) beam and refocus the fundamental (outer) beam to measure a reference ionization yield without the signal beam (*N*_*R*_). The differential ionization yield modulation (δ_N_) is obtained as δ_N_ = *N*_*S*_/*N*_*R*_−1. This differential measurement cancels out the noise originating from the power fluctuation, providing an accurate characterization even for an unstable laser source.

**Figure 6:**
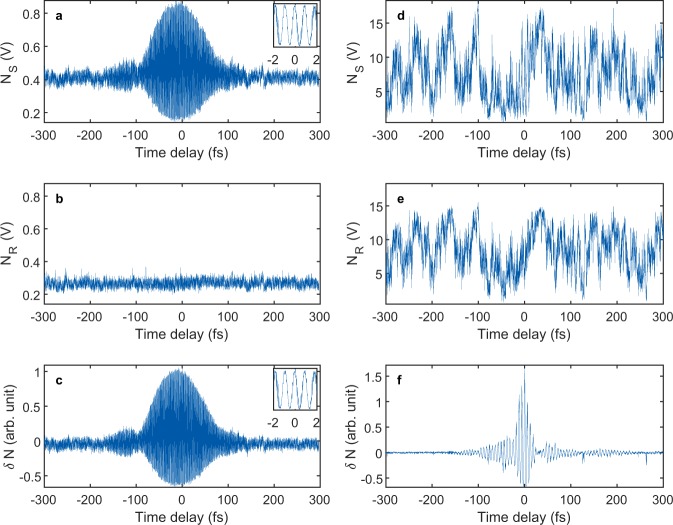
Differential measurements of the stable (266 nm, left) and extremely unstable laser sources (1800 nm, right). (**a**,**b**) Ionization yields obtained using the 266 nm source at the first target N_S_ (**a**) and at the second one N_R_ (**b**) shown in Fig. 5. (**c**) Ionization yield modulation δ_N_ obtained using (**a**) and (**b**). Insets in (**a**) and (**c**) show the ionization yield modulation from −2 fs to 2 fs. (**d**,**e**) Ionization yields N_S_ (**d**) and N_R_ (**e**) obtained using the 1800 nm source. (**f**) Ionization yield modulation δ_N_ obtained using (**d**) and (**e**) (see Methods for the calculation of the ionization yield modulation).

In the following experiments, the advantage of the differential measurement is demonstrated using two laser sources, one stable (266 nm) and the other unstable (1800 nm) (see the Methods section for more details on the light sources). The root-mean-square (RMS) power fluctuation of the 266 nm source was 2.7%, exhibiting decent stability. The 1800 nm source was extremely unstable with energy fluctuations of 9% RMS. Considering that the energy fluctuation of an ordinary commercial Ti:sapphire laser is approximately 1%, the 1800 nm source was very unstable. The ionization yield obtained from two current measurements (N_S_ and N_R_) is shown in Fig. 6 for both cases. While the ionization yield (N_S_) using the 266-nm pulse clearly shows the modulation near zero time delay (Fig. 6a), the ionization yield (N_S_) obtained with the unstable 1800-nm pulse does not clearly show the modulations due to the power fluctuations (Fig. 6d). The corresponding differential ionization yield modulations δ_N_ obtained using the reference ionization yields N_R_ shown in Fig. 6b,e are shown in Fig. 6c,f. While there are no significant changes in the case of the 266-nm pulse, the quality of the signal for the 1800-nm pulse is significantly improved. These measurements indicate that the TIPTOE method can be applied even for extremely unstable sources when the differential measurement is implemented.

**Figure 7:**
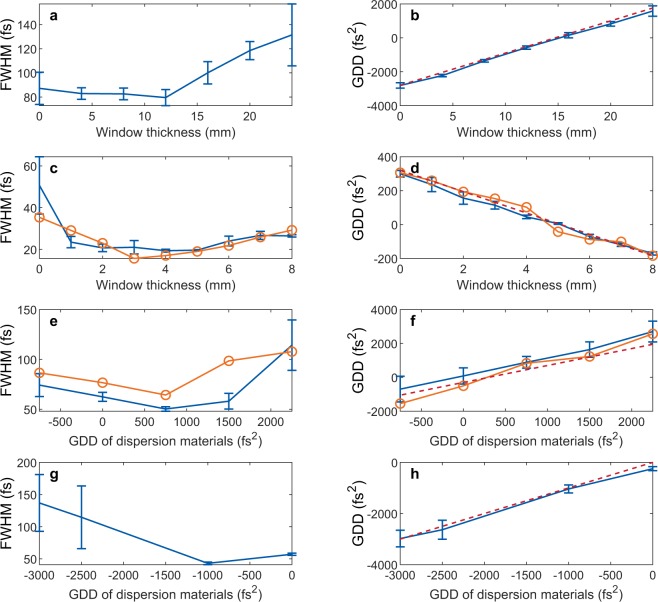
Durations and GDDs obtained with the TIPTOE method. (**a**,**c**,**e**,**g**) The duration (FWHM) measured by TIPTOE as a function of GDDs for 266 nm (**a**), 1800 nm (**c**), 4000 nm (**e**) and 8000 nm (**g**). The pulse durations measured by the FROG method are shown with lines with orange circles for 1800 nm (**c**) and 4000 nm (**e**). (**b**,**d**,**f**,**h**) The GDDs measured by TIPTOE (blue solid line) and calculated from the refractive index of the windows (red dashed line) for 266 nm (**b**), 1800 nm (**d**), 4000 nm (**f**) and 8000 nm (**h**). The GDDs measured by the FROG method are shown with lines with orange circles for 1800 nm (**d**) and 4000 nm (**f**). The offset of the calculated GDD is adjusted for the best fit. (See Methods for the error bar).

For the experimental demonstration of the TIPTOE method at various central wavelengths, pulses are measured under different dispersion conditions using various light sources (266 nm, 1800 nm, 4 μm and 8 μm), as shown in Fig. 7. For all experiments, the intensity of the pulse was kept at the level of 10^12^~10^13^ W/cm^2^ to maintain the higher nonlinearity of ionization, as shown in Fig. 1. The 266-nm beam obtained from sum frequency generation using a BBO crystal was reflected 12 times (6 pairs) on chirped mirrors (Ultrafast innovations) to impose a negative GDD of −1800 fs^2^. Then, a positive GDD was added using multiple quartz windows up to the thickness of 24 mm. The pulse durations and the GDDs are summarized in Fig. 7(a) and 7(b). The pulse duration decreases at the beginning and increases as the glass thickness increases. The pulse durations are longer than the transform-limited duration (50 fs) for the entire GDD range due to the higher-order dispersion. The measured third- and fourth-order dispersions were −5.5 × 10^3^ fs^3^ and 8 × 10^6^ fs^4,^ respectively. The measured GDD as a function of the glass thickness presented in Fig. 7b is in good agreement with the expected GDD. The temporal profiles and the spectra of the 266 nm pulses are shown in Supplementary Fig. S1. These measurements show the applicability of the TIPTOE method in the UV range.

**Figure 8:**
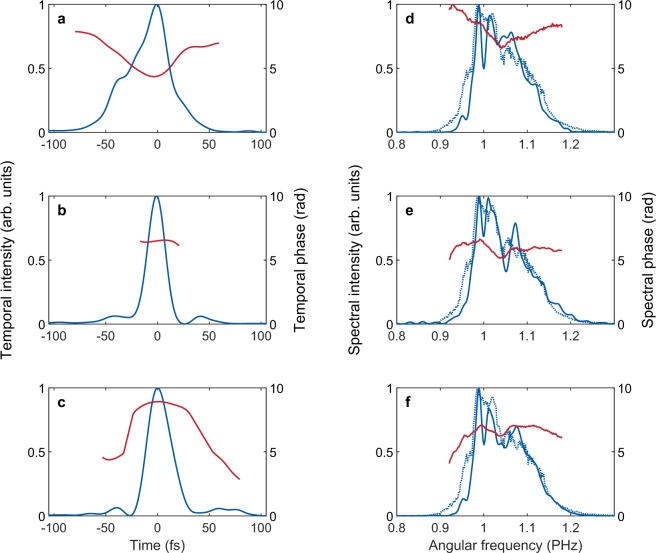
Temporal and spectral profiles of the 1800 nm pulses measured by the TIPTOE method. (**a**–**c**) Temporal intensities (solid blue lines) and phases (solid red lines) measured by TIPTOE are shown for the positively chirped (280 fs^2^) (**a**), chirp-free (10 fs^2^) (**b**), and negatively chirped (−170 fs^2^) (**c**) pulses. (**d**–**f)** Spectral intensities (solid blue lines) and spectral phases (solid red lines) measured by TIPTOE are shown for (**a**–**c**) with the spectrum measured by a spectrometer (dotted blue lines).

For the unstable 1800 nm pulse source, we applied the differential measurements. The pulse duration and the GDD values are summarized in Fig. 7c,d. The dispersion was controlled by placing 0 to 8 mm thick windows in the beam. The GDD decreases as we increase the window thickness at this wavelength. The pulse duration is compared with the values measured by the second harmonic FROG technique. The two results obtained with TIPTOE and FROG show a similar trend. The minimum pulse durations retrieved were 19 fs for the TIPTOE and 16 fs for the FROG technique, as presented in Fig. 7c. The retrieved GDD values are well matched for the two measurements. The temporal and spectral profiles are shown in Fig. 8 for the 280 fs^2^ (positively chirped), 10 fs^2^ (chirp-free) and −170 fs^2^ (negatively chirped) cases. The temporal and spectral phases follow quadratic curves due to the second-order dispersion imposed, confirming the accuracy of the TIPTOE measurement.

Similar measurements were carried out for 4000 nm pulses. As the wavelength increases, the beam size increases at the focus; thus, the intensity decreases. A short-focal-length mirror (f = 5 cm) was used to maintain a sufficiently high intensity (~10^13^ W/cm^2^) to ionize air molecules. The pulse durations and GDDs measured using the single channel measurement setup are summarized in Fig. 7e,f and compared with the results obtained using second harmonic FROG. The dispersion is controlled by calcium fluoride and sapphire plates that impose a negative GDD at 4000 nm. The shortest pulse duration (chirp-free) is 65 fs in both cases, and it increases as the dispersion increases. The GDD values measured by the TIPTOE and the FROG methods are well matched with the expected value calculated from the refractive index of materials. The temporal profiles and the spectra of the pulse are shown in Supplementary Fig. S2.

The TIPTOE technique is also applied for an 8000 nm pulse. The pulse contains spectral components from 6–10 μm. The dispersion is changed through calcium fluoride and zinc selenide crystals that impose a negative GDD. We used a 2.5-cm focal-length mirror with a f-number of f/2.8 to maintain the required peak intensity for ionization with the given pulse energy (40 μJ). The ionization yield modulation is obtained from the differential measurement. The pulse durations and GDDs for 8000 nm pulses are given in Fig. 7g,h. The pulse duration was 40 fs at the chirp-free condition. The GDD values are well matched with the expected values calculated by the refractive index.

For the 8000 nm pulses, the intrinsic fourth order dispersion (FOD) was very large (3 × 10^6^ fs^4^). Thus, the minimum pulse duration was obtained for negative GDD values. The temporal profile and the spectrum of the pulse are shown in Supplementary Fig. S3. The temporal and spectral phases are flat when the pulse duration is minimal. For a negative chirp, the temporal and spectral phases become convex up. For the positive chirp, the phase becomes slightly convex down. These measurements confirm the applicability of the TIPTOE method in the IR wavelength range.

## Discussion

Because the TIPTOE technique utilizes the extreme nonlinearity of ionization, it can be applied for the temporal characterization of femtosecond laser pulses over a broad spectral range. One of the requirements for the TIPTOE measurement is that the pulse energy in the fundamental arm should be high enough for ionization. A pulse energy of 1 uJ was sufficient to implement the TIPTOE method for a 79 fs pulse at 266 nm with a long focal length (25.4 cm, f-number of 48) mirror. However, a pulse energy of 40 μJ was required to see an ionization signal for a 48 fs pulse at 8000 nm with a tight focusing (2.5 cm, f-number of 2.8). The required energy can be reduced by further reducing the f-number of a focusing optics. Alternatively, one can use different gases that have lower ionization potentials. Thus, the TIPTOE method can be generally applied for the temporal characterization of amplified laser pulses.

The temporal shape of a laser pulse is extracted from an ionization modulation in the TIPTOE method. Because ionization occurs multiple times for a multicycle laser pulse, the ionization yield modulation may not directly represent the temporal shape of the test pulse if the pulse duration is much longer than the transform-limited duration. In this work, we used a simple reconstruction method to correct this multicycle effect in which the spectral amplitude is obtained from a separately measured spectrum. It is shown that this approach becomes inaccurate as the pulse duration is much longer than the transform-limited duration. The reconstruction error for the duration and GDD is estimated to be below 5% when τ < 4τ_TL_. An efficient reconstruction algorithm that mitigates this limitation will be developed in a future work.

We demonstrated the applicability of the TIPTOE technique over a broad spectral range using laser pulses at different wavelengths. These results support the applicability of the TIPTOE technique for a multi-octave laser pulse which has been shown theoretically^16^. The applicability of the TIPTOE method for a multi-octave laser pulse should be experimentally verified using a single multi-octave pulse in the future.

In summary, we demonstrated the universality of the TIPTOE technique for a broad wavelength range in the multicycle regime. We showed that the temporal profile of an original pulse can be found from the ionization yield modulation through a simple reconstruction process. The pulse durations and GDDs were in very good agreement with the expected values calculated from the refractive indexes of the material used for the dispersion control. The results obtained at 1800 nm and 4000 nm were in good agreement with those obtained with a second harmonic FROG. These measurements confirm the applicability of the TIPTOE method in the multicycle regime over a broad wavelength range.

## Methods

### Light sources

#### 266 nm source

The 266 nm pulses were obtained by sum frequency generation mixing an 800 nm laser pulse (from a Ti:sapphire laser) with its second harmonic. The beam energy for the measurement was 1.6 μJ and was focused with a 25.4-cm mirror (f-number of 48). The shot-to-shot power fluctuation (2.7% RMS) was estimated with a photodiode.

#### 1800 nm source

The 1800 nm pulse is an idler beam obtained from an optical parametric amplifier (TOPAS, Light conversion). It is compressed using a stretched hollow core fiber system (Few-cycle.com). The FROG method is implemented using a 25-μm BBO crystal.

#### 4000 nm source

The 4000 nm pulses are produced via differential frequency generation (DFG) obtained from a 180-μm LGSe crystal using the signal and idler beams generated from the TOPAS. The FROG method is implemented using a 200-μm AgGaS_2_ crystal.

#### 8000 nm source

The 8000 nm pulses are generated via a DFG beam obtained from a 500-μm-thick GaSe crystal using the signal and idler beams generated from the TOPAS.

#### Other details

To estimate the ionization yield, a current was measured by two metallic plates connected to a current amplifier. A bias voltage of 500 V was applied between the two plates. For the pulse durations and the GDDs shown in Fig. 7, the error bar shows the standard deviation of 5 measurements (266, 1800, and 8000 nm) or 10 measurements (4 um). The ionization yield modulation for the differential measurement was calculated using the ionization yield at the first target (*N*_*S*_) and the second target (*N*_*R*_). Since the amount of the ionization yield *N*_*R*_ would not be exactly the same as *N*_*S*_, *N*_*R*_ is calibrated using the linear function (i.e., *N*_*R*_^′^ = *c*_*R*_*N*_*R*_ + *b*_*R*_). The constants *c*_*R*_ and *b*_*R*_ are determined so that the ratio *N*_*S*_/*N*_*R*_^′^ obtained without the signal pulse becomes unity. Then, the ionization yield modulation is obtained as δ_N_ = *N*_*S*_/*N*_*R*_^′^−1.

## Supplementary Information

Supplementary Information
