# Supplementary material for: Temporal characterization of femtosecond laser pulses using tunneling ionization in the UV, visible, and mid-IR ranges
Source: Sci Rep. 2019 Nov 5;9:16067. doi: 10.1038/s41598-019-52237-y (PMC6831698; doi:10.1038/s41598-019-52237-y)
Supplement: Supplementary file 1 — Supplementary Information [file 41598_2019_52237_MOESM1_ESM.docx]

Fig. S1. Temporal and spectral profiles of the 266 nm pulse measured by the TIPTOE method. (a-c) Temporal intensities (blue solid line) and phases (red solid line) measured by TIPTOE are shown for the positively-chirped (2000 fs^2^) (a), shortest pulse (-1100 fs^2^) (b), and negatively-chirped (-2500 fs^2^) (c) conditions. (d-f) Spectral intensities (blue solid line) and spectral phases (red solid line) measured by TIPTOE are shown for (a-c) with the spectrum measured by a spectrometer (blue dotted line).

 Fig. S2. Temporal and spectral profiles of the 4000 nm pulse measured by the TIPTOE method. (a-c) Temporal intensities (blue solid line) and phases (red solid line) measured by TIPTOE are shown for the positively-chirped (2100 fs^2^) (a), shortest pulse (600 fs^2^) (b), and negatively-chirped (-1800 fs^2^) (c) conditions. (d-f) Spectral intensities (blue solid line) and spectral phases (red solid line) measured by TIPTOE are shown for (a-c) with the spectrum measured by a spectrometer (blue dotted line).

Fig. S3. Temporal and spectral profiles of the 8000 nm pulse measured by the TIPTOE method. (a-c) Temporal intensities (blue solid line) and phases (red solid line) measured by TIPTOE are shown for the positively-chirped (0 fs^2^) (a), shortest pulse (-1000 fs^2^) (b), and negatively-chirped (-2500 fs^2^) (c) conditions. (d-f) Spectral intensities (blue solid line) and spectral phases (red solid line) measured by TIPTOE are shown for (a-c) with the spectrum measured by a spectrometer (blue dotted line).

| Duration of the signal pulse (fs) | Duration of the reconstructed signal pulse (fs) | Error (%) | GDD of the signal pulse (fs^2^) | GDD of the Reconstructed signal pulse (fs^2^) | Error (%) |
| --- | --- | --- | --- | --- | --- |
| 113.69 | 104.71 | -8.57 | -1000.00 | -989.24 | -1.08 |
| 102.90 | 97.47 | -5.57 | -900.00 | -892.58 | -0.82 |
| 92.18 | 88.70 | -3.92 | -800.00 | -792.62 | -0.92 |
| 81.56 | 79.29 | -2.86 | -700.00 | -692.63 | -1.05 |
| 71.08 | 69.52 | -2.25 | -600.00 | -593.49 | -1.08 |
| 60.83 | 59.69 | -1.90 | -500.00 | -493.77 | -1.25 |
| 50.92 | 50.09 | -1.66 | -400.00 | -393.93 | -1.52 |
| 41.62 | 41.07 | -1.32 | -300.00 | -294.40 | -1.87 |
| 33.42 | 33.20 | -0.66 | -200.00 | -196.90 | -1.55 |
| 27.35 | 27.25 | -0.38 | -100.00 | -99.19 | -0.81 |
| 25.00 | 25.00 | 0.00 | 0.00 | -0.65 | n/a |
| 27.35 | 27.25 | -0.38 | 100.00 | 98.89 | -1.11 |
| 33.42 | 33.24 | -0.56 | 200.00 | 197.15 | -1.43 |
| 41.62 | 40.96 | -1.60 | 300.00 | 292.98 | -2.34 |
| 50.92 | 50.83 | -0.19 | 400.00 | 398.00 | -0.50 |
| 60.83 | 60.10 | -1.21 | 500.00 | 496.13 | -0.77 |
| 71.08 | 72.39 | 1.80 | 600.00 | 602.55 | 0.43 |
| 81.56 | 80.95 | -0.75 | 700.00 | 700.06 | 0.01 |
| 92.18 | 92.45 | 0.30 | 800.00 | 805.80 | 0.73 |
| 102.90 | 97.67 | -5.35 | 900.00 | 899.69 | -0.03 |
| 113.69 | 105.86 | -7.40 | 1000.00 | 999.22 | -0.08 |

**Table S1. Duration and GDD values for the original signal and reconstructed signal pulses shown in Fig. 3a.**
